# Fatigue strength of bovine articular cartilage-on-bone under three-point bending: the effect of loading frequency

**DOI:** 10.1186/s12891-017-1510-8

**Published:** 2017-04-04

**Authors:** H. Sadeghi, D. M. Espino, D. E. T. Shepherd

**Affiliations:** Department of Mechanical Engineering, University of Birmingham, Birmingham, B15 2TT UK

**Keywords:** Articular cartilage, Bending, Failure, Frequency, Strength, Three-point

## Abstract

**Background:**

The objective of this study was to determine the influence of loading frequency on the failure of articular cartilage-on-bone specimens under three-point bending.

**Methods:**

In this study, cyclic three-point bending was used to introduce failure into cartilage-on-bone specimens at varying loading frequencies. Sinusiodally varying maximum compressive loads in the range 40–130 N were applied to beam-shaped cartilage-on-bone specimens at frequencies of 1, 10, 50 and 100 Hz.

**Results:**

The number of cycles to failure decreased when loading frequency increased from normal and above gait (1 and 10 Hz) to impulsive loading frequencies (50 and 100 Hz). It was found that 67 and 27% of the specimens reached run-out at loading of 10,000 cycles at frequencies of 1 and 10 Hz, respectively. However, 0% of the specimens reached run-out at loading frequencies of 50 and 100 Hz.

**Conclusion:**

The results indicate that increasing the loading frequency reduces the ability of specimens to resist fracture during bending. The findings underline the importance of the loading frequency concerning the failure of articular cartilage-on-bone and it may have implications in the early onset of osteoarthritis.

## Background

Every joint in the body is subjected to cycles of loading. The function of the articular cartilage is to enable joint surfaces to have low friction movement, with a surface roughness of 80–170 nm [1], and to transmit these loads from one body segment to another [2]. The average person takes approximately 2 million steps per year. Thus, a lower limb joint undergoes 1 million loading cycles during this time. Age has been suggested to be the main factor which predisposes cartilage to damage due to mechanical loading [3]. The lack of blood vessels and cells that can repair significant tissue defects limits the ability of cartilage to repair damage [4].

Although the mechanisms responsible for osteoarthritis remains poorly understood, factors such as obesity and heritable genetics have been suggested to be involved in the progression of the disease [5]. Previous studies [6] have suggested that vigorous physical activities such as frequent bending and lifting are risk factors for osteoarthritis. Such an association implies that mechanical fatigue could also be a factor in the development/progression of osteoarthritis. Freeman et al. [7] suggested that a fatigue mechanism may be associated in the progression of osteoarthritis. Weightman et al. [3, 8], Kempson et al. [9] and Simon et al. [10] have studied compressive, tensile and shear fatigue effects on articular cartilage, respectively. All of these studies have provided data, which demonstrate the progression of damage to cartilage under cyclic mechanical loading.

Rapid heel-strike rise times during gait have been implicated in the onset of osteoarthritis [11]. Cartilage is typically subjected to loading with a heel-strike rise time of 100 to 150 ms [12]. A subset of the population with heel-strike rise times from 5 to 25 ms has been identified as being linked to the onset of osteoarthritis [13]. The timing of these heel-strikes correspond to loading frequencies of 3–5 Hz for normal and up to 90 Hz for rapid heel-strike rise times [14].

Implication of rapid heel-strike in the onset of osteoarthritis is in addition to the link between cartilage failure and mechanical overload of a joint during vigorous physical activities such as heavy manual labour [15]. A previous study [16] has also found evidence suggesting that high internal compressive joint forces by leg muscles during activities which involve deep knee bending maybe a risk factor in osteoarthritis. Therefore, large internal joint forces, applied at frequencies associated with rapid heel-strike rise times might further predispose cartilage to damage during bending, however, this is not currently known. Previous studies [17–19] have suggested that the frequency of loading of a joint might be important with respect to the possibility of damage to articular cartilage.

In the current study, cyclic three-point bend tests have been used to determine the influence of the loading frequency and maximum load on the fatigue strength of articular cartilage-on-bone at loading frequencies corresponding to normal gait (1 Hz), above normal gait (10 Hz) and impulsive/traumatic loading rates (50 and 100 Hz).

## Methods

### Cartilage-on-bone specimens

Humeral heads from bovine shoulder joints were obtained from an established supplier (Dissect supplies, King’s Heath, Birmingham, UK). Joints were from skeletally mature cows which were approximately 24 months old. Bovine tissue was used because it is an established model for human articular cartilage [20]. Furthermore, the uniform surface of the bovine humeral head reduces thickness variability of the specimens, which is beneficial for quantitative experiments [21]. Animals had been slaughtered approximately 48 h previously and kept in refrigerated storage. Upon arrival in the laboratory, the humeral articular surfaces of the joints were examined with the naked eye with the aid of India ink (Loxley Art Materials, Sheffield, UK) to ascertain that they had no damage or degenerative changes [22]. Joints were then wrapped in tissue paper, saturated in Ringer’s solution (Sigma-Aldrich, Dorset, UK), sealed in plastic bags and stored at −40 °C. Prior to testing, joints were thawed at room temperature and cartilage-on-bone specimens were obtained from the humeral head of a joint. Such freeze-thaw treatment does not alter the mechanical properties of cartilage [23, 24] or bone [25].

Rectangular shaped samples, measuring approximately 33 mm × 8 mm along their surface and 4 mm in depth, were cut from the joints. Cartilage-on-bone samples were obtained from the central region of the humeral head. This location was selected because it has a flat surface and is in the centre of the contact region of the joint. Articular cartilage located in this position has been shown to undergo maximum deformation in the humeral head [26].

### Mechanical testing

**Fig. 1:**
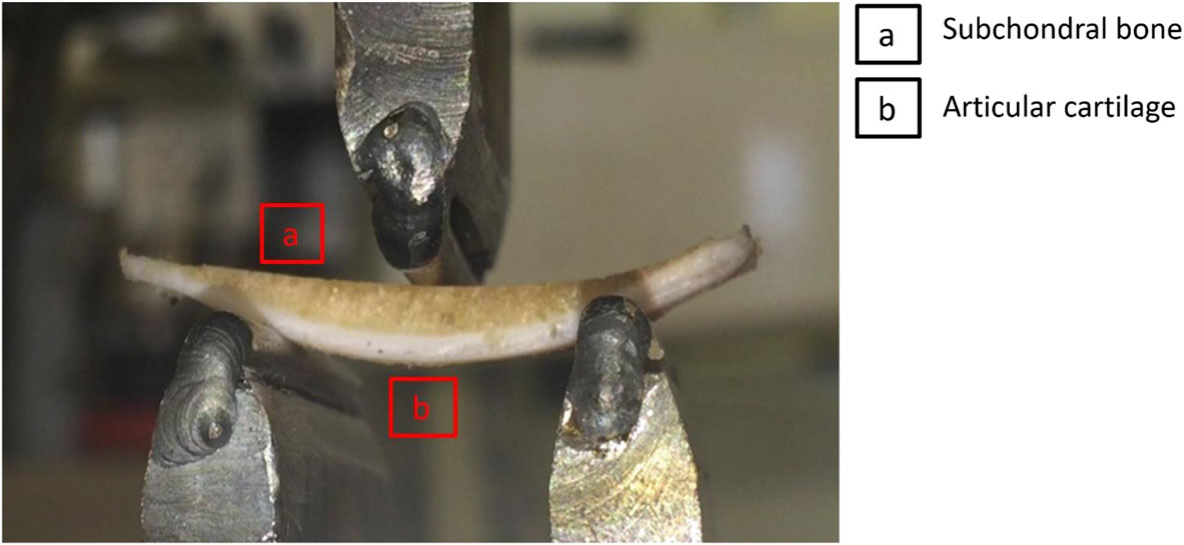
Three-point bend test rig with a cartilage-on-bone specimen in the starting test position

Specimens were subjected to a dynamic three-point bend test, using a Bose ElectroForce ELF3300 materials testing machine (Bose corporation, Minnesota, USA; now, TA instruments, New Castle, DE, USA), operated under the control of WinTest 4.1 software. An aluminium test rig was designed and manufactured to hold the sample and apply the loads (Fig. 1). The lower test rig consisted of two supports (20 mm apart) that attached to the base of the testing machine. The contact radii of the loading supports were 1.5 mm, consistent with a previous study of bone [27]. The upper test rig, attached to the actuator of the testing machine, comprised a bar with a 1.5 mm radius at the contacting end. The specimen was placed on the supports so that the lower supports were in contact with cartilage and the upper roller, which was used to apply the load, was in contact with the subchondral bone (Fig. 1). The length of the specimens varied between 32 and 36 mm, providing overhangs of between 6 and 8 mm on each side of a sample [28]. specimens were under saline irrigation during testing [29].

**Fig. 2:**
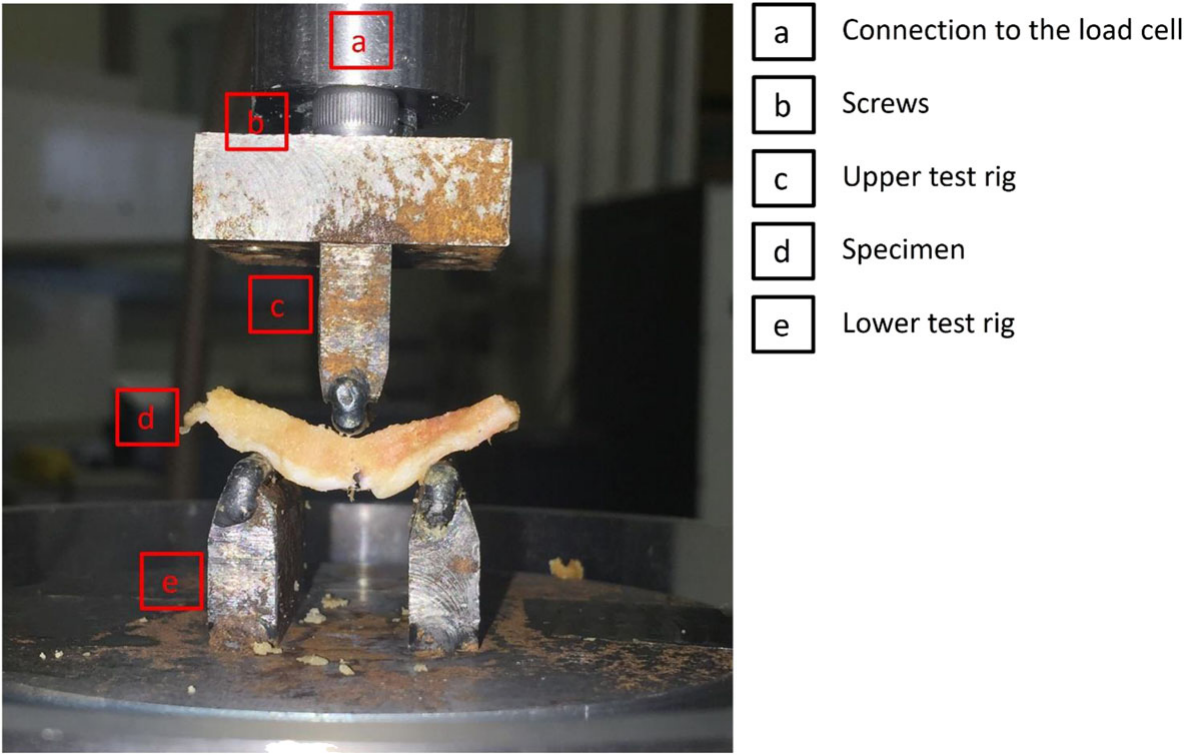
Upper part of the rigid aluminium test rig was tightly attached to the connector to the load cell of the testing machine using three screws. Lower supports were welded to a steel plate. Lower test rig was fixed during testing

The upper test rig included the roller and a rigid aluminium box that was attached to a connector to the load cell using three screws. The lower test rig comprised of two roller supports welded to a steel plate to provide a firm support under dynamic loads (Fig. 2). The testing machine was equipped with a Bose 1010CCH-1 K-B load cell (Bose corporation, Minnesota, USA; now, TA instruments, New Castle, DE, USA), and capable to carry out ± 3 kN dynamic load, at 0 to 100 Hz frequency. The upper part of the test rig was controlled by the software and was capable of travelling ± 12 mm in vertical direction; the lower part was fixed in position during testing.

Testing involved the application of up to 10,000 cycles of sinusoidally varying compressive force at loading frequencies of 1, 10, 50 and 100 Hz [14, 30]. A total of 120 cartilage specimens, obtained from 10 joints, were subjected to the cyclic three-point bend tests. Ten sinusoidally varying compressive force ranges were used for testing. Sinusoidal force ranges started at 4–40 N with the maximum force increased by 10 N up to a force range of 13–130 N; the ratio of maximum to minimum force was 10. These values were chosen based on preliminary tests. The associated peak stresses (*σ*_*max*_) of the selected peak loads (*P*) during cyclic three-point bending tests were calculated from:

1 $$ {\sigma}_{max}=\frac{3 PL}{2 b{h}^2} $$

where *L* is the length, *b* is the width and *h* is the depth of test samples [31]. Therefore, specimens experienced a range of maximum flexural stresses between 15 and 50 MPa. Each test was repeated on three separate specimens. A different specimen was used for every test. Testing continued until complete fracture of the specimen or run out of 10,000 cycles. If a specimen reached 10,000 cycles and it did not fail it was considered to have reached run-out.

After testing specimens that reached run-out were immersed in Ringer’s solution for 30 min, to ensure that the cartilage returned to its original thickness [32]. India ink was applied on the cartilage surface. A visual inspection of individual specimens was undertaken and the cartilage surface was examined. Each specimen was then photographed using a DSC-R1 Cyber-shot© digital camera (10MP, 5 × Optical Zoom) 2.0″ (Sony Corporation, 6-7-35 Kitashinagawa, Shinagawa-ku, Tokyo, Japan).

## Results

**Fig. 3:**
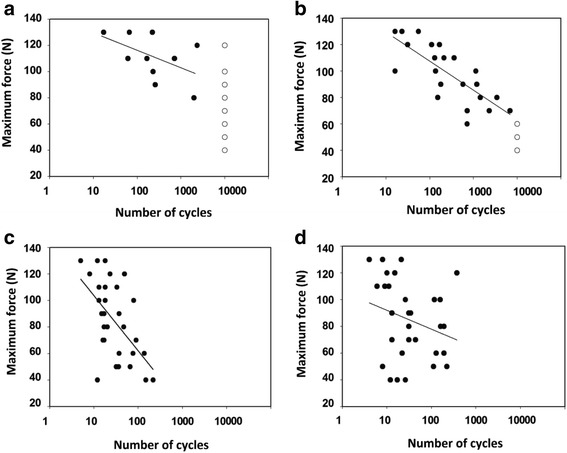
Maximum force plotted against the number of cycles to failure, with the number of cycles on a logarithmic scale (base 10) at loading frequencies of 1 Hz (**a**) 10 Hz (**b**) 50 Hz (**c**) and 100 Hz (**d**). specimens that failed were plotted with (●) and specimens that reached run-out were plotted with (○). Details of the regression curves are included in Table 1

Figure 3 shows the results of the cyclic three-point bend tests of cartilage-on-bone specimens in which the *F-N*-curve for each frequency was plotted. Each data point represents one cartilage-on-bone specimen. The number of cycles to failure decreased with increasing maximum force for all frequencies tested. This relationship can be described using individual logarithmic curve fits in the form:

2 $$ S= A\left( ln(N)\right)+ B $$

where *S* is maximum force, *N* is number of cycles to failure and *A* and *B* are constants.

The number of cycles to failure decreased significantly (*p* < 0.05) with the increasing maximum force at loading frequencies of 10 and 50 Hz. The correlation was not significant (*p* ≥ 0.05) at 1 or 100 Hz. The corresponding *p* and *R* ^*2*^ values are provided in Table 1.

**Table 1:** Constants from the curve fit correlations of Fig. 3

Curve fit	Loading frequency (Hz)	A (SE)	B (SE)	*p*	*R* ^2^
(a)	1	−5.6 (3.4)	142 (19.0)	0.12	0.25
(b)	10	−9.6 (1.8)	152 (10.5)	<0.001	0.77
(c)	50	−20 (4.6)	150 (14.5)	<0.001	0.52
(d)	100	−6 (4.2)	106 (15.6)	0.24	0.04

*SE* is the standard error of the coefficients *A* and *B. R2* is a squared correlation coefficient and shows how well the lines fit the data points. If *p* < 0.05 it indicates that the line is statistically significant

From the specimens that were subjected to three-point bend tests at a loading frequency of 1 Hz, 20 out of 30 specimens reached run-out of 10,000 cycles (67%; Fig. 3a). The number of specimens that reached run-out at a loading frequency of 10 Hz was 8 out of 30 (27%; Fig. 3b). However, none of the samples reached run-out at loading frequencies of 50 or 100 Hz (Fig. 3c and d). The number of cycles to failure were in the ranges of 5 to 217 and 6 to 374 at frequencies of 50 and 100 Hz, respectively.

**Fig. 4:**
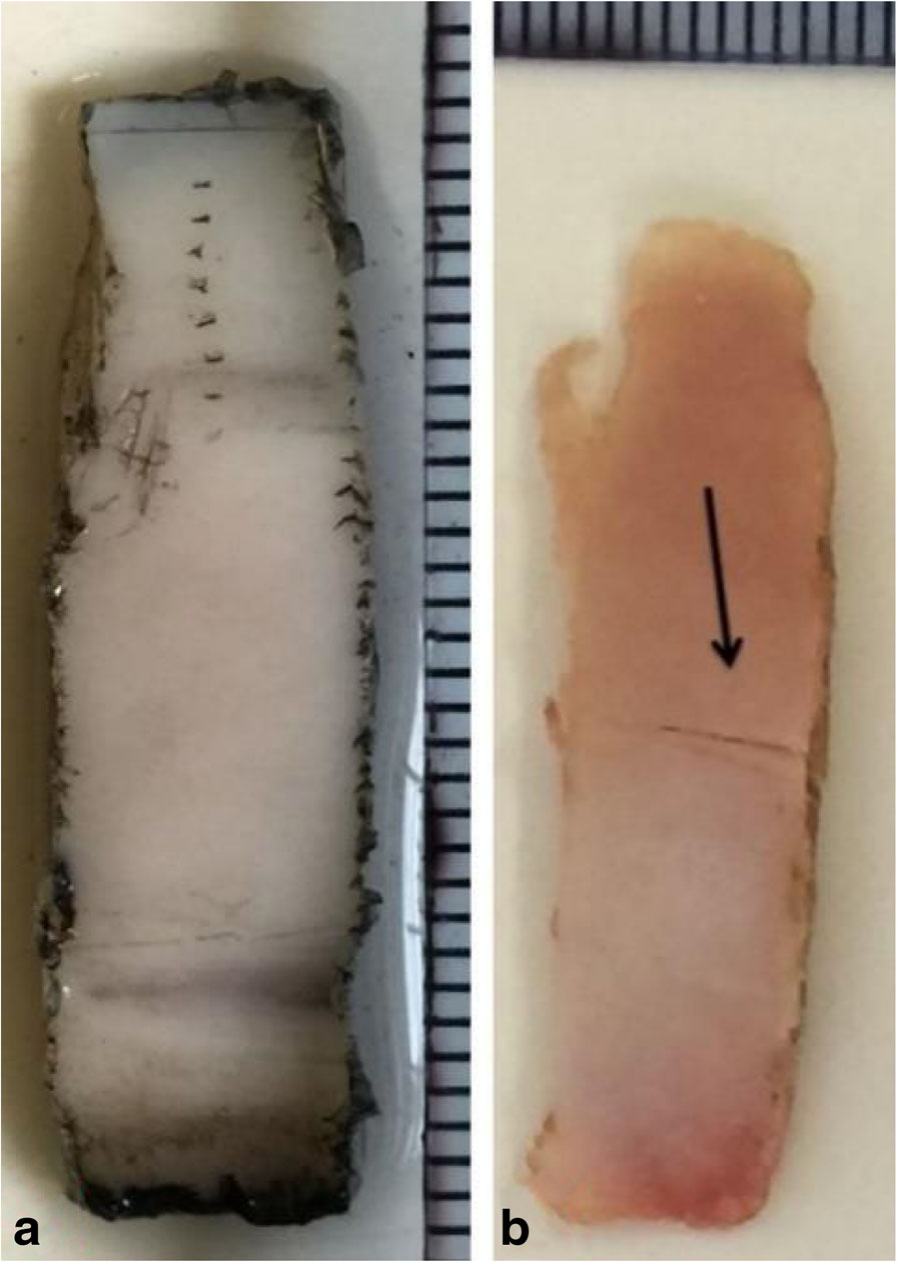
Cartilage surface crack observed on specimens that reached run-out at loading frequencies of 1 Hz and 10 Hz (**a**) Top view of the selected specimens after run-out was reached at a loading frequency of 1 Hz and (**b**) 10 Hz. Both specimens in these images were loaded in the 6–60 N

Images of two samples that reached run-out, which had been loaded between 6 and 60 N (maximum flexural stress of 23 MPa) at loading frequencies of 1 and 10 Hz are shown in Fig. 4a and b, respectively. Through qualitative assessments after each test, it was observed that four out of eight of the specimens, which reached run-out at a loading frequency of 10 Hz, had surface cracks at the centre of the specimens. However, no signs of damage were observed for specimens, which completed run-out, at a loading frequency of 1 Hz (Fig. 4).

## Discussion

This study has used sinusoidally varying compressive cyclic force to produce failure in cartilage-on-bone specimens under three-point bend tests. The number of cycles to failure decreased with increasing maximum force. The logarithmic curve fits of the *F-N*-curves were statistically significant for loading frequencies of 10 and 50 Hz, however, this relationship was not found to be significant at loading frequencies of 1 and 100 Hz. Increasing the loading frequency resulted in increased failure for a given load. It was found that 67% of the specimens reached run-out at frequency of 1 Hz, 27% at 10 Hz, but 0% at frequencies of 50 and 100 Hz.

The effect of loading frequency on fatigue strength under bending has previously been carried out on bone only. An in vitro study of bovine cortical bone [33] found that increasing the frequency of loading from 30 to 125 Hz decreased the number of cycles required to produce fracture by a factor of three to four. They suggested that the acceleration of fatigue strength tests with increasing the loading frequency is a result of increased strain rate. Another similar study [34] reported that the effect of loading frequency on bending fatigue strength of bone below 30 Hz is negligible. Loading frequencies above 30 Hz resulted in a shorter fatigue life. This is consistent with the present study where cartilage-on-bone specimens subjected to three-point bending decreased in the number of cycles to failure when frequency increased.

The variation in the number of cycles to failure with increasing loading frequency from physiological (1 and 10 Hz) to impulsive loading frequencies (50 and 100 Hz) could correspond to before/after the glass transition in cartilage [14]. Articular cartilage has been shown to behave as a viscoelastic material from below gait relevant frequencies such as 0.001 to 1 Hz [35] up to an impulsive loading frequency of 92 Hz [14]. Fulcher et al. [14] showed that storage modulus increased with increasing frequency, but it was asymptotic above 20 Hz, attributed to a glass transition, while the loss modulus remained constant. The dependence of storage modulus on frequency, in which storage modulus increases but then levels out to a plateau, is characteristic of a material undergoing a glass transition [36]. However, to date, there are no data available on the variation of mechanical properties of bone with increasing loading frequency. Therefore, it is difficult to distinguish between the effect of frequency on bone and cartilage as related to failure.

Results from this study showed that increasing the loading frequency towards impulsive frequencies (50 and 100 Hz) resulted in more specimen failures. This is in agreement with a study on bovine cartilage under impact testing in vitro showing that when high rate impact was applied on the surface of cartilage-on-bone explants this caused severe damage to the tissue [37]. Burgin et al. [38] also showed that increased energy of deformation (per unit volume) occurred in cartilage with increased stress and strain rates.

This current study found that 10,000 loading cycles at a frequency of 10 Hz with an induced stress of 23 MPa caused cracks on half of the cartilage surfaces of specimens that reached ‘run-out’. However, the same peak load and number of cycles at 1 Hz did not create any cracks. This observation is consistent with previous findings that following 10,000 cycles of compressive stress in the range of between ~ 3 and 7.5 MPa, through an indenter, produced surface cracks on cartilage [30]. It should be noted that both this current study and previous study by Sadeghi et al. [30] tested cartilage-on-bone. The physical behaviour of cartilage when on and off-bone was suggested to be different [39] because of the restraining effects of the underlying bone to cartilage [40, 41]. The cartilage-bone interface has been reported to predispose cartilage-on-bone specimens to the formation of cracks [42]. This interface is characterized by fibrils approximately perpendicular to the articular surface [43]. Repetitive shear stresses developed at the cartilage-bone interface have been suggested to produce cracks and splits similar to that observed in osteoarthritic cartilage [44] particularly under impulsive loading [19, 45].

In this study, the number of cycles required to cause cartilage failure decreased with increasing maximum force. This is consistent with tensile fatigue failure for off-bone cartilage during cyclic loading [9, 46]. Kempson et al. [46] extrapolated from their data that 30 year-old patellar cartilage should not fail in vivo until the age of 200 years. The results from this study showed that increasing the frequency used for cyclic loading lowered the tensile strength of cartilage. The same conclusion has been stated when tensile fatigue failure of cartilage, leading to the prediction of advancing failure with age under tensile loading [47, 48]. Weightman et al. [49] observed changes on the surface of the femoral head (identified using India ink) which occurred after 90,000 compression cycles. Another study of cartilage-on-bone also revealed that cyclic loading disrupts the tissue and the severity of the damage increased with increasing load and number of cycles [50]. Compression fatigue fracture strength of lumbar functional spinal units is also shown in vitro, where incorporating specimen-specific and load-specific parameters into a Wöhler analysis resulted in linear relationships for *F-N*-curves [51]. However, the number of cycles used by Huber et al. [51] was greater than this study reaching up to 300,000 cycles.

Flexural stresses of cyclic three-point bend tests in this study were calculated, using Eq. 1, to be in the range 15 to 50 MPa. Stresses used in this study are comparable to a previous cyclic compressive study of cartilage-on-bone specimens [52], which reported that the mean fracture strength of bovine articular cartilage was 35.7 MPa. Cyclic tensile loading studies, instead, have found experimental damage to occur in human cartilage with the number of cycles up to 1.5 million and stresses in the range of 1–3 MPa [53] or 97,200 cycles under stresses that averaged 3.2 MPa in vitro [54]. The differences in the results could be explained by the fact that failure was defined as the fracture of bovine cartilage and its underlying bone in the current study, whereas a previous study defined failure as the rupture of off-bone human cartilage specimens under tension [53]. Further, in our study bovine specimens were used, as compared to the human cartilage used by Bellucci et al. [53]. Lower failure stresses for cartilage have also been reported in the lower region of 8 MPa under static compressive loading [55, 56]; however, failure was defined as merely large cracks on the surface of the cartilage not failure of the full cartilage depth and its underlying subchondral bone.

Articular cartilage damage, in the form of fissures and fragments, has been observed clinically [57] and it is similar to those produced experimentally by single and repetitive impact loads [58]. A threshold of 15–25 MPa was reported to cause subchondral bone fracture and surface fissures on the cartilage surface [59]. None of these studies have assessed the influence of the loading frequency of impacts on the failure stresses reported. Other single impact studies [60, 61] have reported the fracture threshold stress of cartilage-on-bone specimens to be 50 MPa, consistent with the largest maximum stress used in this study.

### Limitations

One of the limitations of this study could be that the specimens used in this study were comprised of two layers, one of cartilage and another of subchondral bone. The cartilage-on-bone specimens were maximum 4 mm in depth. The cartilage layer of the bovine humeral head is typically 0.6–1.8 mm [62]. Therefore, the thickness of subchondral varies between 2.2 and 3.4 mm. In this context, it should be remembered that the stiffness of subchondral bone [63] is several orders of magnitude higher than that of the uncalcified cartilage [29]. Therefore, the ratio of cartilage to bone might influence the overall stiffness of the specimens and consequently the strain of the tissue under bending. However, it should be noted that all specimens were tested to failure, and that samples were arbitrarily selected for a given test procedure. Thus, the increased propensity to failure observed at higher frequencies is very much expected to be a consequence of an intrinsic weakness at higher frequencies of loading.

Another limitation of this study is that Eq. 1 provides an approximation of the stresses experienced by the specimens during testing. This is because according to ASTM D7774-12 [31], Eq. 1 is applied to isotropic, homogeneous and beam shaped specimens with uniform thickness. However, specimens used in this study were layered and varied in thickness along the length. Stresses experienced by the cartilage layer may also be higher than calculated due to the positioning of the bending neutral axis.

The duration of each test frequencies varied between 2 h 47 min and 100 s at frequencies of 1 and 100 Hz, respectively. Thus, the specimen dynamic deformation might have been accompanied by creep deformation. One possibility was to allow samples to recover between each set of loading frequencies, in proportion to the duration of loading [64]. However, previous fatigue tests on cartilage scaffolds, have been carried out up to 100,000 continuous unconfined compression cycles [65]. Moreover, the increased loading time to which cartilage was exposed at lower frequencies, may have expected to lead to additional damage of specimens tested. However, the opposite trend was observed, more damage ensued at higher frequencies of loading (i.e., shortest duration of loading). Thus, if anything our results may under-predict the effect of frequency on failure.

## Conclusion

During bending the number of cycles to failure decreased with increased maximum force at all loading frequencies. Independent of load, the number of cycles to failure under bending decreased when the loading frequency increased from normal and above gait (1 and 10 Hz) to impulsive loading frequencies (50 and 100 Hz). Furthermore, the proportion of specimens reaching run-out of 10,000 cycles under three-point bending decreased from 67 to 27% at physiological loading frequencies of 1 and 10 Hz, respectively, to 0% at 50 and 100 Hz. Therefore, an increased loading frequency predisposes articular cartilage on-bone to damage during bending.
